# Radiological Assessment of Atypical Femoral Fractures: A 10‐Year Experience Across Two Centres

**DOI:** 10.1155/joos/6097091

**Published:** 2026-08-24

**Authors:** Aongus O’Brolchain, Ian Hughes, Richard Steer, Alfred Phillips, Chen-I. Lin, Zander Englebrecht, Katherine Griffin

**Affiliations:** ^1^ Department of Medicine, Griffith University, Nathan, Queensland, Australia, health.qld.gov.au; ^2^ Department of Endocrinology, Gold Coast University Hospital, Southport, Queensland, Australia, health.qld.gov.au; ^3^ Department of Research, Gold Coast University Hospital, Southport, Queensland, Australia, health.qld.gov.au; ^4^ Department of Orthopaedic Surgery, Gold Coast University Hospital, Southport, Queensland, Australia, health.qld.gov.au; ^5^ Department of Radiology, Gold Coast University Hospital, Southport, Queensland, Australia, health.qld.gov.au

**Keywords:** atypical, fracture, transverse

## Abstract

**Objective(s):**

Atypical femoral fractures (AFFs) are stress or insufficiency fractures occurring in the femoral shaft associated with long‐term osteoporosis treatment with antiresorptives. Expeditious diagnosis of AFF is important, as ongoing treatment with antiresorptives is then contraindicated. AFFs can occur bilaterally, either simultaneously or sequentially; prompt diagnosis is pivotal to ensuring appropriate surgical and medical management. The American Society for Bone and Mineral Research (ASBMR) has devised diagnostic criteria based on history and distinctive radiographic features that are qualitative and subjective. Recently, the Sydney AFF Score (SS) has been devised which is more quantitative and objective, based on the age of the patient and two specific radiographic measurements. This study aimed to assess the global performance, sensitivity and specificity of the SS in this cohort. Secondary aims were to assess interobserver agreement in the assessment of the ASBMR criteria and SS measurements, the accuracy of radiological reporting and the utility of ICD‐10 codes for diagnosis of AFF.

**Design:**

Retrospective observational study of patients presenting with AFFs or ‘typical’ femoral fractures (TFFs) as diagnosed by an expert panel.

**Setting:**

Two teaching hospitals in Queensland, Australia. Primary outcomes measure(s): (1) diagnosis of AFF using the SS; (2) measuring sensitivity and specificity of each ASBMR criterion for AFF; (3) accuracy of radiology reporting; (4) accuracy of ICD‐10 codes in identifying AFF.

**Methods:**

Adult patients aged 18 years or older presenting with low‐energy mechanism, subtrochanteric fractures between January 2012 and February 2022 were screened from the medical records using ICD‐10 codes. A total of 46 AFFs were identified following expert panel review, with 38 TFFs in a comparator group. The sensitivity and specificity of both the SS (cortical width ≥ 5 mm at lesser trochanter, femoral neck width ≤ 37 mm and age < 80 years) and each of the ASBMR criteria was assessed. The degree of interobserver variability was measured using the Kappa statistic.

**Results:**

Out of 869 fractures, 46 AFFs were identified following independent expert panel review by a 2/3 majority, and a comparator group of 38 female patients was selected from the remaining 72 TFFs. The major radiological ASBMR criteria demonstrating highest diagnostic performance were transverse orientation (91.4%, 89.1%), minimal comminution (99.2%, 63.9%) and periosteal reaction (89.4%, 96.4%). The ASBMR criteria showed good interobserver agreement (Kappa), transverse orientation (0.72), minimal comminution (0.64) and local periosteal reaction (0.72), whereas the agreement with the anthropometric indices using the SS was less robust: lateral cortex ≥ 5 mm (0.25) and femoral neck width ≤ 37 mm (0.66). A ‘modified’ SS was created by incorporating the ‘transverse’ criterion. Of the AFFs, 13.2% of radiology reports in medical records referenced atypical or drug‐related fracture.

**Conclusions:**

Qualitative indices of the ASBMR outperformed the SS. Incorporating transverse fracture orientation into the SS and using more specific landmarks for measurements may yield superior diagnostic value and help to mitigate subjectivity in the assessment of radiographs. This study confirms that AFFs are under‐reported in pelvic X‐rays.

**Summary.**
• This study examined radiological tools for diagnosing atypical femoral fractures in two Australian centres. We found incorporating fracture angle improved accuracy of the Sydney Score. Findings highlight under‐recognition of AFFs by radiologists and support more objective diagnostic approaches to guide patient care.

## 1. Introduction

The prevalence of osteoporosis continues to increase worldwide. In 2023, there was one osteoporotic fracture every 2.7 min in Australia [1]. Antiresorptive treatment remains the cornerstone of osteoporosis management, and bisphosphonates have remained the first‐line treatment for nearly 30 years [2]. In 2010, the United States Food and Drug Administration approved a newer antiresorptive: a receptor activator of nuclear factor kappa‐Β ligand (RANKL) inhibitor denosumab. Both classes of antiresorptive are effective treatments and prevent ‘typical’ osteoporotic femoral fragility fractures (TFFs). However, the rare complication of ‘atypical’ femoral fractures (AFFs) is highly associated with bisphosphonate treatment, particularly long term. AFF occurs at a frequency of approximately 6 per 100,000 person years [3], of which 93% are in women [4]. Schilcher estimated the relative risk (RR) of AFF to be 55 (95% CI 39, 79) in women using bisphosphonate, which increased to 126 (55, 288) for treatment greater than 4 years [3]. Amongst bisphosphonate users, women were at greater risk than men, RR = 3.1 (1.1, 8.4) [3]. AFF has also been associated with excessive femoral bowing and Asian ethnicity [4, 5].

TFFs are characterised by a predilection for the proximal femur (femoral neck and between the greater and lesser trochanters), are unilateral in nature (on the side of minimal trauma), are comminuted and have an acute fracture angle. These ‘insufficiency’ fractures usually occur at the weight‐bearing, medial cortex of the femoral neck and shaft. Additionally, TFFs are not usually portended by prodromal pain [6].

In contrast, AFFs are phenotypically distinct as reflected in qualitative radiological criteria such as the fracture being distal to the lesser trochanter and proximal to the supracondylar flare, having a transverse orientation involving the lateral cortex with localised endosteal or periosteal thickening (‘beaking’), and there being no or minimal comminution. Additionally, AFF are often bilateral, and commonly associated with a period of prodromal thigh pain and functional limitation. As is the case with TFF, AFF are associated with minimal trauma. ‘Incomplete’ AFFs involve the lateral cortex only and continue medially from the site of initiation in a transverse orientation. ‘Complete’ AFFs continue further to breach the medial cortex, often with a characteristic ‘medial spike’ [7]. ‘Stress’ fractures, such as those caused by mechanical overload in athletes, have a similar appearance to AFF, but the pathophysiological mechanism of AFF in patients treated with antiresorptives is not as well‐understood [8]. However, it is known that bisphosphonates inhibit osteoclast activity, leading to a decrease in bone remodelling, an accumulation of bone microdamage and, hence, potential for stress fracture [9].

**FIGURE 1:**
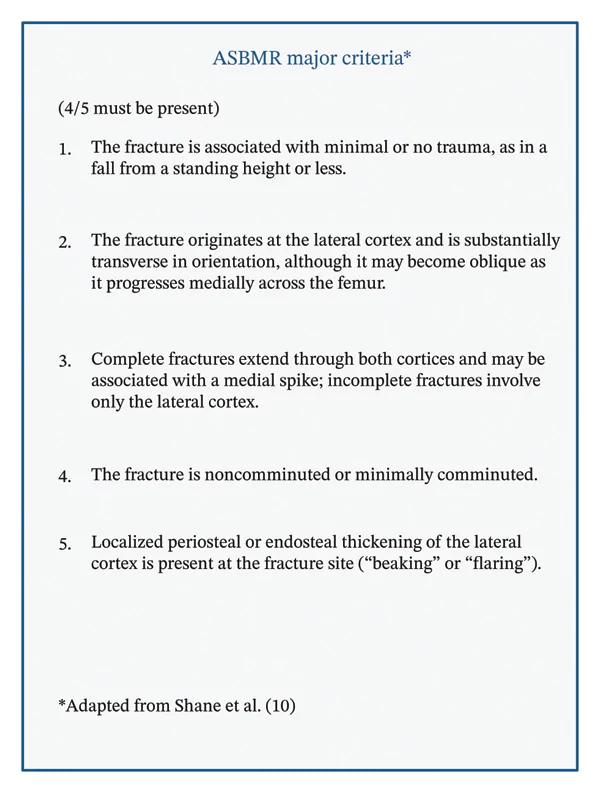
ASBMR criteria for AFF.

Based on the fracture resulting from minimal trauma and the radiological criteria noted above, the American Society for Bone and Mineral Research (ASBMR) devised, in 2010, and revised in 2014, a diagnostic rubric for AFF diagnosis (Figure 1) [7, 9]. To diagnose AFF, the fractures must occur between the lesser trochanter and supracondylar flare and must meet at least four out of the five criteria of being associated with minimal trauma, and the four major radiological criteria, as shown in Figure 1. However, several studies have demonstrated interobserver variability in interpretation of these qualitative radiological criteria [10]. Other studies have suggested, based on unrelated pelvic and femoral X‐rays, that AFFs are underdiagnosed [11, 12], particularly incomplete AFFs [13], possibly due to the rarity of AFF or their being seen primarily only within the orthogeriatric subspecialty. These observations have led to attempts to create more objective, quantitative diagnostic criteria. The Sydney AFF Score (SS) was devised by Crouch et al. (2021) to expedite and facilitate the diagnosis of AFF in female patients using age and measurements from anterior–posterior (AP) radiographs [3]. Specifically, one point is scored for age ≤ 80 years, femoral neck width < 37 mm and lateral cortical width at the lesser trochanter ≥ 5 mm. The scores are summed, and if ≥ 2, AFF is diagnosed. Crouch et al. a reported a sensitivity of 73% and specificity of 70% for AFF diagnosis [3].

This study aimed to test the sensitivity and specificity of the SS in our cohort of patients with femoral fractures distal to the lesser trochanter and proximal to the supracondylar flare and compare these to diagnoses of AFF by ASBMR criteria. We were also interested to see if the SS could be improved by inclusion of other quantitative predictors. Secondary aims were to estimate interobserver agreement for both ASBMR and SS‐based diagnoses, to determine the proportion of radiograph reports correctly diagnosing AFF and to assess the accuracy of ICD‐10 coding in those reports.

## 2. Methods

**FIGURE 2:**
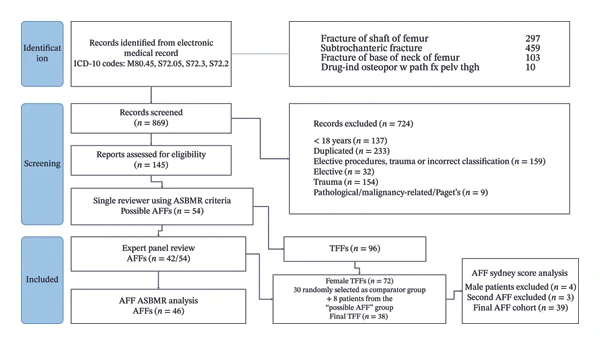
Flow diagram of patient screening, exclusion, adjudication and inclusion in analyses. Femoral shaft fractures identified through ICD‐10 coding underwent manual radiographic screening and expert adjudication according to ASBMR criteria. Comparator TFF cases were randomly selected following exclusion of AFFs.

The study was conducted at the Gold Coast University and Robina Hospitals on the Gold Coast, Queensland, Australia (ethics approval (LNR) HREC/2022/QGC/84798; approved 20 April 2022). As this was a retrospective review of existing medical records and imaging, the Human Research Ethics Committee waived the requirement for individual informed consent in accordance with the National Statement on Ethical Conduct in Human Research (Sections 2.3.9–2.3.10). This study builds upon our previously published analysis of AFF cases in the same cohort [14]. A total of 869 femoral shaft fractures of patients presenting between January 2012 and March 2022 were identified from the electronic medical record using ICD‐10 codes. In accordance with the ASBMR criteria (Figure 1), all fractures proximal to the lesser trochanter and distal to the supracondylar flare (including neck of femur and intertrochanteric fractures) were excluded following review of radiographs [9]. Mechanism of injury was ascertained after reviewing individual medical charts, and all high‐energy traumatic femoral fractures were excluded (greater than a fall from standing height). Additionally, all patients under 18 years of age were excluded. The remaining subtrochanteric and diaphyseal and femoral shaft fractures (M80.45—*Drug induced osteoporosis with pathological fracture, pelvic region and thigh*, *Fracture of base of neck of femur* S72.05, *Fracture of shaft of femur* S72.3, *subtrochanteric fracture* S72.2) were included (Figure 2). All pathological fractures relating to malignancy or other disease of bone mineralisation (e.g. Paget’s disease) were excluded. As the SS has been validated in female patients only, male patients were excluded from analyses involving the SS. However, male patients were retained for analyses involving ASBMR criteria, given that these criteria are not sex‐specific. All ASBMR‐based diagnostic performance estimates therefore include both male and female patients, whereas SS analyses are restricted to females.

One hundred and forty‐five fractures met inclusion criteria (Figure 2). Following the procedure used by Crouch et al. in developing the SS [3], a single independent investigator reviewed all radiographs and identified possible AFFs based on the ASBMR criteria. The comparator cohort was randomly selected to achieve an approximately 1:1 case–control ratio, consistent with the original SS development study. This design enabled efficient expert radiographic review while maintaining balanced estimation of diagnostic discrimination. Inclusion of all remaining TFFs would have substantially increased reviewer workload with little expected improvement in precision for this exploratory validation study.

Three expert clinicians (two orthopaedic surgeons and a radiologist), the observers, were then tasked to provide a definitive diagnosis of AFF based on ASBMR criteria, consistent with our previous study [14]. Prior to commencement of image review, the three observers participated in a calibration session during which representative AFF and TFF radiographs were reviewed and discussed to standardise interpretation of the ASBMR criteria and radiographic measurements before independent assessment. Blinded to the demographic and clinical details (other than trauma being of low energy) of the patients, the observers reviewed the images independently in random order. A definitive diagnosis of AFF was made based on fractures meeting at least 3 out of 4 of the ASBMR radiological criteria by a 2/3 majority of the observers. Any ‘possible’ AFFs adjudicated as TFFs by the observers were added to the comparator group. The same observers were then provided the radiographs again in random order and asked, in a standardised data collection tool, if each ASBMR criterion was met and to quantitatively report the radiological measures required for the SS.

This study adheres to the STROBE (Strengthening the Reporting of Observational Studies in Epidemiology) guidelines for case–control studies. A completed STROBE checklist is provided in the Supplement.

### 2.1. SS

The SS is composed of three criteria: lateral cortical width (LCW) ≥ 5 mm at the lesser trochanter, femoral neck width (FNW) < 37 mm and age ≤ 80 years. For each criterion met, 1 point is accrued, with a score of ≥ 2 leading to an AFF diagnosis [3]. The score is not validated for use in male patients, given the extremely low incidence among males and due to sexually dimorphic anthropometry [3]. In accordance with the SS study procedure [3], where there was gross deformity or excessive comminution, LCW and FNW were measured in the contralateral limb. In cases of bilateral, simultaneous femoral fractures, measurements were performed on the least deformed limb. In cases of sequential fractures, the index presentation only was included for the purposes of measurement.

LCW at the lesser trochanter and FNW were measured and recorded as continuous and as dichotomous variables (< or ≥ 37 mm for FNW and ≥ or < 5 mm for LCW) [3]. We used the recommended cut‐off of a score of ≥ 2 for diagnosing AFF using the SS [3]. We also estimated the optimal cut‐off based on our data.

Exploratory post hoc analyses were undertaken to assess whether incorporation of additional radiographic features, including transverse fracture orientation, improved diagnostic discrimination.

### 2.2. Radiology Reporting

For radiology reports, all cases of AFF as determined by the expert panel were included. Contemporaneous radiology reports for all 46 AFFs were reviewed using the electronic medical record and the hospital imaging programs Inteleviewer and Agfa Enterprise PACS. References in the reports to ‘pathological’, ‘bisphosphonate‐related’, ‘antiresorptive’ and ‘atypical’ fractures were recorded. Additionally, recommendation (or absence thereof) for further imaging of the contralateral limb was documented.

### 2.3. Diagnostic Classification

The accuracy of ICD‐10 diagnostic classification was assessed by comparing the documented diagnostic codes following expert adjudication of AFF. The diagnosis of AFF was assumed following report of ICD‐10 code M80.45—*Drug induced osteoporosis with pathological fracture, pelvic region and thigh*.

### 2.4. Statistical Analysis

Statistical analyses were performed using Stata17 and 18 (College Station, TX, USA). Age of patients in the TFF and AFF groups was summarised by mean and standard deviation (SD) and compared using a *t*‐test. Sensitivity and specificity for the ASBMR definition of AFF were calculated for each observer using the definitive AFF/TFF diagnosis as the gold standard. Similarly, sensitivity and specificity were calculated for each individual radiological ASBMR criterion and compared to assess relative importance. Criterion 3, ‘incomplete fractures involve only the lateral cortex’, is conditional on the fracture being incomplete and so was not included in these analyses. Sensitivity and specificity of the SS for diagnosis of AFF at the recommended threshold of 2 were calculated. Area under the receiver operating characteristic curve (AUC) was used to assess the overall diagnostic performance of the SS and any modification to the SS. The AUC was compared between diagnostic modalities using the roccomp command of Stata utilising the nonparametric algorithm of Delong et al. [15]. For any modified SS, the optimal cut point was estimated using the method of Liu [16], which maximises the product of sensitivity and specificity, and sensitivities and specificities calculated.

As Crouch et al. used logistic regression to create the SS, we used logistic regression to assess the effect of adding transverse nature of the fracture to the dichotomous variables, LCW ≥ 5 mm, FNW < 37 mm and age > 80 years, to the model.

Interobserver agreement for categorical variables, such as AFF diagnosis by ASBMR criteria or SS, was quantified using the Fleiss kappa statistic (Kappa) as observers were assumed to represent a random sample of expert clinicians. Qualitative interpretation of Kappa was based on the Landis and Kock benchmark scale and the 95% confidence interval of the Kappa estimate [17].

Meta‐analysis, using the metan command of Stata, was used to assess overall diagnostic accuracy across observers and heterogeneity between observers in relation to sensitivities, specificities and AUCs. The I^2^ statistic estimates the proportion of variation due to heterogeneity where values greater than 50% were considered evidence of substantial heterogeneity. If heterogeneity was less than substantial, estimates could be reliably pooled across observers. Sensitivities and specificities of any modified SS were compared to the original SS using the exact McNemar test.

## 3. Results

Fifty‐four ‘possible’ AFFs and 96 TFFs were identified by the single independent reviewer using ASBMR criteria. Following expert adjudication by the three observers, 46 AFFs were identified, with the remainder added to the comparator TFF cohort to total 38.

Secondary outcome: diagnostic classification accuracy.

Five out of 46 AFFs (10.87%) were correctly classified as AFF using the ICD‐10 code as *M.80.45*—*Drug-induced osteoporosis with pathological fracture Pelvic region and thigh*.

The mean (SD) age for patients with AFF and TFF was 77.9 years (12.4) and 74.7 years (14.8), respectively (*p* = 0.23). Four patients were male, all of whom had a diagnosis of AFF. Given the very small number of male patients and the absence of validation of the SS in males, formal subgroup analyses were not undertaken. Nevertheless, male AFF cases fulfilled ASBMR diagnostic criteria and were retained in analyses involving radiographic criteria independent of sex‐specific anthropometric measurements.

### 3.1. ASBMR Criteria

In assessing the ASBMR criteria, all 46 AFFs including those from males were used. The sensitivity and specificity of each radiological parameter used in the ASBMR criteria for each observer, in addition to the overall, ASBMR criteria met diagnosis of AFF, are shown in Table 1. These sensitivities and specificities were calculated based on satisfying ASBMR criteria according to each observer when compared to the expert gold‐standard AFF designations or diagnoses. In general, AFF diagnosis based on ‘ASBMR criteria met’ had very high specificity and high sensitivity with ‘transverse orientation’ on its own showing very high sensitivities and specificities. ‘Minimal comminution’ had higher sensitivities than specificities, while ‘periosteal reaction’ had higher specificities than sensitivities. Observers, 1, 2 and 3 identified 8, 10 and 11 fractures as being incomplete, respectively, with 8, 8 and 9 identified as involving the lateral cortex.

**TABLE 1:** American Society for Bone and Mineral Research (ASBMR) criteria.

Criterion (*n* = 81)	Observer	Sensitivity %	95% CI	Specificity %	95% CI
Transverse orientation	1	97.7	(88.0, 99.9)	91.9	(78.1, 98.3)
	2	95.3	(84.2, 99.4)	81.1	(64.8, 92.0)
	3	90.9	(78.3, 97.5)	94.6	(81.8, 99.3)

Minimal comminution	1	97.7	(88.0, 99.9)	67.6	(50.2, 82.0)
	2	100	(91.6, 100)	73.0	(55.9, 86.2)
	3	100	(92.0, 100)	51.4	(34.4, 68.1)

Periosteal reaction	1	86.4	(72.6, 94.8)	97.3	(85.8, 99.9)
	2	97.6	(87.4, 99.9)	91.9	(78.1, 98.3)
	3	84.1	(69.9, 93.4)	100	(90.5, 100)

ASBMR criteria met^1^	1	88.6	(75.4, 96.2)	100	(95.5, 100)
	2	95.2	(83.8, 99.4)	89.2	(74.6, 97.0)
	3	75.0	(59.7, 86.8)	100	(90.5, 100)

*Note:* Table 1: Sensitivities and specificities with 95% confidence intervals (CI) of the ASBMR criteria (including males).

^1^ASBMR criteria met denotes fractures meeting 4/5 radiological criteria irrespective of whether they were deemed AFFs by observers.

The Fleiss Kappa inter‐rater agreement observed for AFF diagnosis by meeting ASBMR criteria was 0.66 (0.53, 0.79), indicating moderate to substantial agreement. This was 0.72 (0.60, 0.85) for ‘transverse orientation’ (substantial to almost perfect) and 0.64 (0.50, 0.79) for ‘minimal comminution’ (moderate to substantial).

### 3.2. Radiology Reporting

Of the 46 definitively diagnosed AFFs, 28.26% (13/46) of radiology reports referenced them as being pathological, bisphosphonate‐related or AFFs. In 23.9% (11/46) of cases, a suggestion was made to image the contralateral limb. However, in seven cases, an intramedullary nail in the contralateral femur obviated the necessity for contralateral imaging (this is because the prophylactic treatment of incomplete or high‐risk contralateral lesions is the placement of an intramedullary nail). Of cases where the fracture was reported as atypical/pathological, specific mention of long‐term bisphosphonate use was included in the request form in 23% (3/13).

### 3.3. SS

**FIGURE 3:**
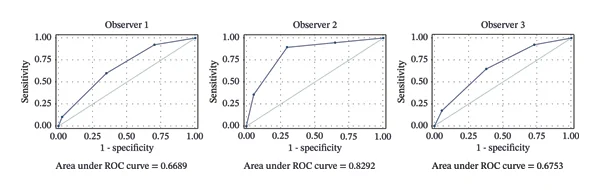
Receiver operating characteristic curves for the Sydney Score for observers 1–3 demonstrating interobserver variability in diagnostic performance.

**FIGURE 4:**
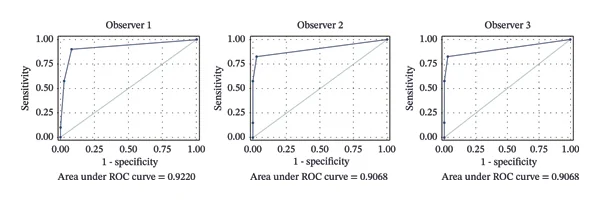
Receiver operating characteristic curves for the modified Sydney Score incorporating transverse fracture orientation for observers 1–3.

Following exclusion of male patients and those patients with multiple fractures, 39 AFFs were included in the analysis of the SS variables, with 38 in the comparator group. The sensitivities and specificities, at the recommended cut‐point of ≥ 2 for each observer, are shown in Table 2. The optimal cut‐point for the SS based on our data was also found to be ≥ 2 for each observer. The AUC for the SS for each observer is also shown in Table 2 and in Figures 2, 3 and 4 as is the pooled AUC following meta‐analysis, 0.73 (95% CI 0.62, 0.84), suggesting a moderate to good discriminative ability [18]. However, the I^2^ = 67.9% indicated a high proportion of total variation in the SS AUC estimate that was due to observer variation rather than the true SS AUC variation. The SS indices, FNW < 37 mm and LCW ≥ 5 mm, were assessed in relation to inter‐rater agreement with FNW having a Fleiss Kappa of 0.66 (0.53, 0.79), demonstrating moderate agreement, and LCW 0.25 (0.09, 0.40), demonstrating slight to fair agreement.

**TABLE 2:** Diagnostic criteria for each method of AFF diagnosis.

Method/observer	AUC (95% CI)	*p* value	I^2^%	Sensitivity %^∗^ (95% CI)	*p* value	I^2^%	Specificity %^∗^ (95% CI)	*p* value	I^2^%	Kappa^∗^ (95% CI)
ASBMR met										
Observer 1	0.938 (0.886, 0.989)			87.5 (73.2, 95.8)			100 (90.5, 100)			
Observer 2	0.920 (0.859, 0.982)			94.9 (82.7, 99.4)			89.2 (74.6, 97.0)			
Observer 3	0.863 (0.792, 0.933)			72.5 (56.1, 85.4)			100 (90.5, 100)			
Pooled	0.912 (0.870, 0.953)		28.7	86.2 (74.1, 98.4)		71.3	99.1 (95.9, 100)		0.1	0.642 (0.505, 0.780)
SS										
Observer 1	0.667 (0.555, 0.783)	0.0001^a^		60.0 (43.3, 75.1)	0.02^a^		64.9 (47.5, 79.8)	0.0002^a^		
Observer 2	0.829 (0.739, 0.919)	0.096^a^		90.0 (76.3, 97.2)	0.69^a^		70.3 (53.0, 84.1)	0.060^a^		
Observer 3	0.675 (0.562, 0.789)	0.0097^a^		65.0 (48.3, 79.4)	0.65^a^		62.2 (44.8, 77.5)	0.0001^a^		
Pooled	0.730 (0.622, 0.837)		67.9	72.5 (53.6, 91.4)		82.2	65.9 (0.567, 0.751)		0	0.582 (0.441, 0.723)
mSS										
Observer 1	0.922 (0.861, 0.982)	3 × 10^−6^ ^b^		90.0 (76.3, 97.2)	0.002^b^		91.9 (78.1, 98.3)	0.010^b^		
Observer 2	0.907 (0.838, 0.976)	0.03^b^		89.7 (75.8, 97.1)	1.00^b^		89.2 (74.6, 97.0)	0.040^b^		
Observer 3	0.907 (0.845, 0.969)	0.0001^b^		82.5 (67.2, 92.7)	0.09^b^		97.3 (85.8, 99.9)	0.001^b^		
Pooled	0.912 (0.876, 0.949)		0.1	88.0 (81.6, 94.4)		0.0	94.3 (89.1, 99.5)		5.0	0.662 (0.526, 0.798)
Transverse										
Observer 1	0.947 (0.896, 0.999)	0.31^c^		97.5 (86.8, 99.9)	0.25^c^		91.9 (78.1, 98.3)	1.00^c^		
Observer 2	0.880 (0.807, 0.953)	0.40^c^		94.9 (82.7, 99.4)	0.50^c^		81.1 (64.8, 92.0)	0.250^c^		
Observer 3	0.923 (0.863, 0.983)	0.54^c^		90.0 (76.3, 97.2)	0.25^c^		94.6 (81.8, 99.3)	1.00^c^		
Pooled	0.924 (0.889, 0.959)		3.3	87.3 (76.3, 98.4)		70.8	99.1 (95.9, 100)		0.1	1.713 (0.583, 0.842)

*Note:* For consistency of comparisons, only females were included in the estimation of statistics.

Abbreviations: ASBMR = American Society for Bone and Mineral Research, AUC = area under the curve, CI = confidence interval, mSS = modified Sydney Score, SS = Sydney Score.

^∗^For ASMBR criteria met, SS ≥ 2, mSS ≥ 1, transverse fracture.

^a^ASBMR compared to SS for the observer listed.

^b^SS compared to mSS for the observer listed.

^c^mSS compared to transverse for the observer listed.

### 3.4. Inclusion of Transverse Fracture Orientation Significantly Improved Model Performance

During the analysis, it was evident that many patients with an SS of ≥ 2 (recommended cut‐off) did not have fractures with transverse orientation, resulting in a number of false positives. Therefore, a ‘modified’ SS (mSS) was tested whereby a dichotomous variable was created, incorporating the qualitative ASBMR criterion that the fracture be ‘substantially transverse in orientation’, in addition to the SS indices (FNW < 37 mm, LCW ≥ 5 mm). The modified score was automatically ‘0’ if the fracture was not substantially transverse as recorded by the reviewer. The AUROC for the mSS for each observer is shown in Table 2 and in Figure 4. The optimal cut‐point for the mSS was found to be ≥ 1 for each observer. The sensitivities and specificities of the mSS at this cut‐point are also shown in Table 2.

For mSS, the pooled AUC was estimated as 0.91 (0.88, 0.95), suggesting very good discriminative ability. The I^2^ = 0.05% indicated essentially no heterogeneity between observers.

**FIGURE 5:**
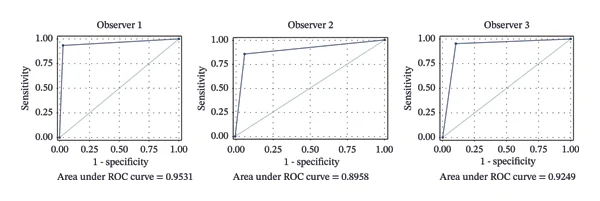
Receiver operating characteristic curves for transverse fracture orientation as an isolated radiographic discriminator of AFF for observers 1–3.

AUC, and sensitivities and specificities between ASMBR criteria met and SS, between SS and mSS, and between mSS and the ‘transverse’ criterion were compared (Table 2). In summary, the SS did not perform as well as individual ASBMR criteria and the mSS with respect to AUC (overall diagnostic performance), specificity and sensitivity. Compared with ASBMR criteria–based diagnosis, the SS demonstrated significantly lower diagnostic performance for observers 1 and 3 (AUC comparison *p* = 0.0001 and *p* = 0.0097, respectively), whereas no statistically significant difference was observed for observer 2 (*p* = 0.096). Incorporation of transverse fracture orientation into the modified SS significantly improved diagnostic performance compared with the SS alone across observers (observer 1, *p* = 3 × 10^−6^; observer 2, *p* = 0.03; observer 3, *p* = 0.0001). Transverse fracture orientation demonstrated high AUC, sensitivity, and specificity, with diagnostic performance comparable to the modified SS (all *p* ≥ 0.25; Table 2). Receiver operating characteristic curves for transverse fracture orientation for observers 1–3 are shown in Figure 5.

## 4. Discussion

This study shows that AFF is under‐recognised by reporting radiologists. Of all fractures identified as AFF by our expert panel, just 28% of the radiology reports documented this diagnosis. This observation underscores the need for increased awareness and more objective diagnostic systems.

The performance of the SS in this cohort (AUC = 0.73) was comparable to that reported in the original study’s validation cohort (AUC = 0.76) [3]. This study demonstrated a high degree of heterogeneity and lack of observer agreement in the measurement and interpretation of radiographic anthropometric indices used in the SS, particularly LCW. The low interobserver agreement observed for LCW measurement (*κ* = 0.25) represents an important practical limitation of the SS and may reduce reproducibility in routine clinical settings. Variability likely reflects ambiguity in anatomical landmarks, radiographic rotation and subjective interpretation of cortical thickening. Standardisation of measurement technique, clearer radiographic definitions, dedicated training or semiautomated measurement approaches may improve reproducibility in future studies. These findings, together with the omission of transverse fracture orientation from the SS, represent key limitations of the tool. Our study reaffirms the high sensitivity and specificity of the ASMBR radiological criteria in the diagnosis of AFF with good interobserver agreement and low heterogeneity. Le Blanc et al. showed that significant discrepancies were evident between reviewers’ ‘gestalt’ impression of a fracture as atypical, one‐third of patients meeting ASBMR criteria were not classified as AFFs, compared to using the ASBMR criteria. In the same study, more than one‐third of patients meeting ASBMR criteria were not classified as AFFs by expert reviewers [18]. In our study, 86% of fractures technically meeting ASBMR criteria were adjudicated as AFF.

Previous studies have demonstrated that, among the ASBMR criteria, the transverse pattern of the fracture from the lateral cortex has high sensitivity and specificity [19]. One study showed a sensitivity of 93.6% and specificity of 95.5%, with the authors also demonstrating that it was associated with the least interobserver variability [10]. The findings of our study are consistent with these previously published results. By incorporating the qualitative ‘transverse’ criterion into the SS, we constructed the modified SS, mSS, which improved the sensitivity and specificity markedly, particularly reducing the number of false positive results. Importantly, transverse fracture orientation alone demonstrated diagnostic performance comparable to that of the modified SS. These findings suggest that transverse fracture orientation functions as an important discriminator of AFF and may explain a substantial proportion of the observed improvement in diagnostic performance when incorporated into the modified SS. In practical terms, these findings support fracture orientation as a key radiographic discriminator that may complement, rather than replace, broader structured diagnostic assessment. Such simplification could enhance clinical usability and reduce measurement variability. However, as this modification was both derived and tested within the same dataset, prospective external validation would be required before recommending streamlined diagnostic algorithms.

The transverse criterion, with its high sensitivity and specificity for diagnosing AFFs, is a good candidate for conversion into a quantitative measure. The traditional AO (Arbeitsgemeinschaft für Osteosynthesefragen) provides a definition of ‘transverse’ as being less than 30° from the perpendicular to the long axis of the diaphysis [8, 20]. Schilcher et al. described measuring the fracture angle from the lateral diaphyseal femoral cortex of the proximal fragment of the fracture. A line is drawn parallel to the lateral cortex (ignoring beaking/lateral cortical focal thickening), and the angle measured to a second line that is drawn parallel to the fracture, coursing medially, to about 1/3 of the way through the width of the femoral neck [8]. Schilcher et al. showed that 95% of AFFs had fracture angles between 69° and 109°, equivalent to between 21° and −19° under the AO definition [8].

Our findings suggest that incorporation of a quantitative measure of transverse fracture orientation may represent a promising component of a future diagnostic framework for AFF. However, because the modified SS was both derived and evaluated within the same dataset, the reported AUC represents apparent rather than externally validated performance and is therefore susceptible to optimism bias. Accordingly, the observed improvement should be regarded as exploratory and hypothesis‐generating. External validation in an independent cohort is required before the modified SS can be recommended for routine clinical use.

A future study is planned to formally evaluate the incorporation of fracture angle into a revised, quantitative diagnostic algorithm for AFFs. Further investigation will require additional independent observers to ensure reliability across multiple raters and imaging modalities, as well as a separate validation cohort to confirm generalisability of findings beyond the initial dataset. The development and prospective testing of a modified scoring system incorporating fracture angle could provide a standardised approach to AFF recognition and mitigate current diagnostic inconsistencies. The structured and geometric nature of fracture angle measurement suggests potential applicability to automated radiographic analysis. However, no artificial intelligence–based evaluation was performed in this study, and these considerations should be regarded as hypothesis‐generating only. Prior studies have explored machine learning approaches to AFF detection [13], but such models require careful validation and may be limited by the small case numbers inherent to rare conditions. Future research should prospectively evaluate whether incorporation of quantitative fracture orientation into automated systems improves diagnostic consistency and reproducibility.

The poor sensitivity of ICD‐10 coding for AFF identification highlights limitations in the use of administrative datasets for epidemiological surveillance and retrospective case ascertainment. Reliance on coding alone may substantially underestimate true disease burden and emphasises the importance of radiographic review when studying rare fracture phenotypes.

As the prevalence of osteoporosis increases, patients receiving antiresorptive treatment will be encountered with increasing frequency both in primary care and in the hospital setting. It is therefore essential that all physicians encountering patients treated with antiresorptives be familiar with potential complications of their use, as prompt investigation may prevent progression from incomplete to complete fracture. A significant proportion of AFFs are bilateral and may occur simultaneously or sequentially [5] Failure to recognise AFFs (incomplete and complete) may thus result in both a failure to remove the underlying cause (antiresorptive treatment) and prevent further fractures by screening for and/or prophylactic management of the contralateral femur, which in high‐risk patients has been shown to be cost‐effective [21].

Strengths of this study include a relatively large sample size given the low incidence of AFFs, adjudication by an interdisciplinary panel of experts and comprehensive, chart‐by‐chart review of all patients presenting with femoral‐shaft fractures at a tertiary centre over more than 10 years. Nevertheless, the cohort remains modest in absolute terms and geographically confined to a single health service in Queensland, Australia. Further studies in larger, ethnically diverse and multinational populations are needed to confirm the generalisability of our findings Inclusion of all femoral shaft/diaphyseal fractures mitigated the risk of selection bias.

This study has limitations that should be considered. For consistency with the original SS development study, the same three expert observers who established the reference diagnosis subsequently performed structured assessment of the ASBMR criteria and SS measurements. This introduces the potential for incorporation and review bias, because the radiographic features used by the expert panel to establish the reference diagnosis also formed the basis of subsequent structured assessment of the ASBMR criteria and SS. Consequently, complete independence between the reference standard and index tests cannot be assumed, and the reported diagnostic performance—particularly for ASBMR‐based approaches—may represent an upper‐bound estimate of performance achievable in routine clinical practice. Although observers were blinded to clinical information and radiographs were re‐presented in random order, residual correlation between the reference standard adjudication and subsequent structured assessment cannot be excluded. Independent external adjudication was not feasible given the rarity of AFF and the requirement for subspecialist musculoskeletal radiographic expertise. Prospective studies incorporating independent reference standard adjudication would provide a more rigorous assessment of diagnostic performance.

Enrichment of AFF and comparator TFF cases facilitated balanced assessment of diagnostic discrimination but does not reflect the true prevalence of AFF in routine practice and may limit external generalisability. Restricting detailed adjudication to fractures initially considered potentially compatible with AFF creates spectrum restriction, as the study population does not represent the full spectrum of femoral fractures encountered in routine clinical practice. Consequently, diagnostic performance estimates may not be directly applicable to unselected populations presenting with proximal or femoral shaft fractures. Although this approach was chosen to mirror development of the original SS and maximise efficiency for a rare condition, external validation in consecutive, unselected fracture cohorts will be necessary to establish generalisability.

Additional limitations include radiographic rotation, which may limit derivation of fracture angle measurements, the retrospective design with potential for missing data and imperfect case ascertainment using ICD‐10 coding. However, inclusion of all femoral shaft fractures for screening mitigated the risk of missed cases, and these limitations are unlikely to fully explain the observed interobserver heterogeneity or the consistent diagnostic strength of transverse fracture orientation.

## 5. Conclusion

This study demonstrates that AFFs remain under‐recognised on routine radiographic reporting, confirms the diagnostic robustness of ASBMR criteria and identifies transverse fracture orientation as a promising radiographic discriminator that warrants prospective evaluation within future quantitative diagnostic algorithms. Failure to identify AFF can potentially result in suboptimal treatment and prophylaxis and highlights the necessity to raise awareness about this rare but potentially preventable condition.

## Author Contributions

Aongus O’Brolchain conceived and designed the study, performed statistical analysis, conducted data extraction and data entry, synthesised tables and figures, and drafted the manuscript.

Ian Hughes contributed to statistical analysis and critically reviewed and edited the manuscript.

Richard Steer, Alfred Phillips, and Chen‐I. Lin performed independent expert radiographic review and contributed to data interpretation.

Zander Englebrecht assisted with data extraction and data entry.

Katherine Griffin contributed to study design, provided senior oversight and reviewed and edited the manuscript.

## Funding

No funding was received for this manuscript.

Open access publishing was facilitated by Griffith University, as part of the Wiley–Griffith University agreement via the Council of Australasian University Librarians.

## Conflicts of Interest

Aongus O’Brolchain, Ian Hughes, Richard Steer, Chen‐I. Lin, Zander Englebrecht and Katherine Griffin declare no conflicts of interest.

## Supporting Information

Additional supporting information can be found online in the Supporting Information section.

## Supporting information


**Supporting Information** . Supporting File 1. Completed STROBE reporting checklist for this retrospective observational study.

## Data Availability

The data supporting the findings of this study are available from the corresponding author upon reasonable request.
