# Supplementary material for: Radiological Assessment of Atypical Femoral Fractures: A 10‐Year Experience Across Two Centres
Source: J Osteoporos. 2026 Aug 24;2026:6097091. doi: 10.1155/joos/6097091 (PMC13501500; doi:10.1155/joos/6097091)
Supplement: Supplementary file 1 [file JOOS-2026-6097091-s001.docx]

STROBE Checklist:

**STROBE Checklist for Case–Control Studies**

| **Item No.** | **Recommendation** | **Page / Section** | **Completed?** |
| --- | --- | --- | --- |
| 1 | Indicate study design in title/abstract; provide structured summary. | Title & Abstract | Yes |
| 2 | Explain scientific background and rationale. | Introduction | Yes |
| 3 | State specific objectives and hypotheses. | Introduction | Yes |
| 4 | Present key elements of the study design. | Methods — Study Design | Yes |
| 5 | Describe setting, locations, and relevant dates. | Methods — Setting | Yes |
| 6a | Eligibility criteria; case/control selection. | Methods — Participants | Yes |
| 7 | Define exposures, outcomes, predictors, confounders. | Methods — Variables | Yes |
| 8 | Explain how study size was chosen. | Methods — Study Size | Yes |
| 9 | Describe methods to assess exposures and outcomes. | Methods — Assessment | Yes |
| 10 | Describe any efforts to address potential bias. | Methods — Bias | Yes |
| 11 | Explain handling of quantitative variables. | Methods — Statistical Analysis | Yes |
| 12a | Describe statistical methods, including confounder control. | Methods — Statistical Analysis | Yes |
| 13a | Report numbers of individuals at each stage. | Results — Flow | Yes |
| 14a | Describe participant characteristics. | Results — Baseline | Yes |
| 15 | Report outcome data for case–control studies. | Results | Yes |
| 16a | Report unadjusted and adjusted estimates with CIs. | Results — Statistical Analysis | Yes |
| 18 | Summarise key results. | Discussion | Yes |
| 19 | Discuss study limitations. | Discussion — Limitations | Yes |
| 20 | Offer overall interpretation considering other evidence. | Discussion — Interpretation | Yes |
| 21 | Discuss generalisability/external validity. | Discussion — Generalisability | Yes |
| 22 | Give source of funding and role of funders. | End of Manuscript | Yes |
